# Comprehensive EST analysis of Atlantic halibut (*Hippoglossus hippoglossus*), a commercially relevant aquaculture species

**DOI:** 10.1186/1471-2164-8-144

**Published:** 2007-06-04

**Authors:** Susan E Douglas, Leah C Knickle, Jennifer Kimball, Michael E Reith

**Affiliations:** 1 Institute for Marine Biosciences,1411 Oxford Street, Halifax, Nova Scotia, B3H 3Z1, Canada

## Abstract

**Background:**

An essential first step in the genomic characterisation of a new species, in this case Atlantic halibut (*Hippoglossus hippoglossus*), is the generation of EST information. This forms the basis for subsequent microarray design, SNP detection and the placement of novel markers on genetic linkage maps.

**Results:**

Normalised directional cDNA libraries were constructed from five different larval stages (hatching, mouth-opening, midway to metamorphosis, premetamorphosis, and post-metamorphosis) and eight different adult tissues (testis, ovary, liver, head kidney, spleen, skin, gill, and intestine). Recombination efficiency of the libraries ranged from 91–98% and insert size averaged 1.4 kb. Approximately 1000 clones were sequenced from the 5'-end of each library and after trimming, 12675 good sequences were obtained. Redundancy within each library was very low and assembly of the entire EST collection into contigs resulted in 7738 unique sequences of which 6722 (87%) had matches in Genbank. Removal of ESTs and contigs that originated from bacteria or food organisms resulted in a total of 7710 unique halibut sequences.

**Conclusion:**

A Unigene collection of 7710 functionally annotated ESTs has been assembled from Atlantic halibut. These have been incorporated into a publicly available, searchable database and form the basis for an oligonucleotide microarray that can be used as a tool to study gene expression in this economically important aquacultured fish.

## Background

Atlantic halibut is a cold-water flatfish native to the North Atlantic that shows excellent potential for production in aquaculture due to its highly prized white flesh. Flatfish have long been a choice food fish, with many members of the group e.g., halibuts, flounders, soles, turbot, and plaice, having great commercial value especially in Asia. With the general worldwide decline in the wild fishery, and the predicted global collapse of all currently fished species by the year 2048 [1], it is crucial that alternatives such as aquaculture be pursued. Investigations into producing flatfish by aquaculture have been underway for the last fifteen to twenty years. Aquaculture production of Japanese flounder, turbot, Atlantic halibut and others has now been successfully achieved, although improvements in efficiency are still clearly required.

Production of Atlantic halibut is relatively recent and is currently underway in Norway, Iceland, Scotland and Canada. Significant hurdles must still be overcome, particularly with regard to judging when to spawn females, reproduction and sex determination [2], nutrition [3,4], and enhancing disease resistance. The application of genomics technologies to thoroughly characterize the biological processes of reproduction, development, nutrition, and immunity promises to improve our knowledge of this poorly understood fish and provide for long-term enhancements in aquaculture production.

Flatfish, members of the order Pleuronectiformes, comprise a biologically interesting group of fish. During development, in a process known as metamorphosis, these fish reorient themselves to lie on one side, the body flattens, and the eye migrates to the other side of the body. This settling of the fish on the side vacated by the migrating eye requires a complex reorganization of skeletal, nervous and muscle tissues [5]. Significant losses due to mortality in the early larval stages, as well as developmental abnormalities such as malpigmentation [6,7], bone deformities [8], and incomplete eye migration [8], have hampered the successful production of flatfish. A better understanding of these processes at the molecular level and the impact that rearing conditions have on survival, metamorphosis, and growth will improve the commercial feasibility of flatfish aquaculture.

Expressed sequence tag (EST) surveys of species of interest provide a great deal of information-rich data on the expressed portion of an organism's genome [9] and are invaluable in comparative genomics [10]. In addition, they can be an important source of microsatellites and single nucleotide polymorphisms (SNPs) that can be used for genetic mapping [11]. EST surveys in teleosts have been performed from both non-normalised cDNA libraries and libraries generated by suppression subtractive hybridisation [12] in order to elucidate genes involved in immunity [13-28], muscle formation [29], endocrinology [30-32], and toxin production [33]. The most highly represented teleost in dbEST is the zebrafish, *Danio rerio*, with over 1.3 million ESTs. Salmonids are also well-represented with over half a millions ESTs. Economically important fish species such as catfish, cod, Japanese flounder, sea bream and sea bass have increasingly been the subject of genomic studies and these species are now represented by several thousand or even tens of thousands of ESTs.

Among the flatfish, considerable effort has been made to determine ESTs for Japanese flounder [13,17,22]. There are currently 8842 ESTs for this species, although most are unannotated. Furthermore, the majority of these sequences arise from non-normalised cDNA libraries or those constructed from immune-stimulated fish; few ESTs have been sequenced from non-immune tissues or different developmental stages of flatfish. For turbot, the other main commercially relevant flatfish, there are no published EST studies and most of the sequences from this species in GenBank are microsatellites or rRNA.

As a first step towards developing genomics tools for Atlantic halibut, a large-scale EST survey was performed, annotations undertaken where possible, and a searchable database set up. Considerable effort has been made to annotate these ESTs and associate them with Gene Ontology (GO) terms to facilitate subsequent microarray analyses. The species-independent structured GO vocabulary [34] is widely accepted and used in most large scale genome annotation projects.

## Results

### cDNA libraries and ESTs

**Figure 1:**
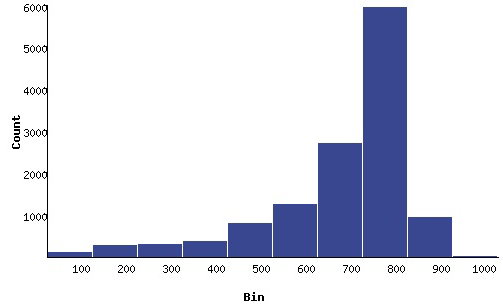
Representation of sequencing read lengths of Atlantic halibut Expressed Sequence Tags (ESTs). Read lengths were binned in 100 base pair (bp) increments. Most of the ESTs fall into the 700–800 bp bin.

Normalized cDNA libraries were constructed from eight Atlantic halibut tissues and five developmental stages. For all libraries, the percentage of clones with inserts was high, ranging from 91 to 98%, with average insert sizes between 1.3 and 1.6 kb (Table 1). After trimming and vector removal, 12675 good sequences were obtained. Average read length after trimming for each library was approximately 600–700 bp with the majority of reads compiled from all 13 libraries being between 700 and 800 bp (Figure 1). Clustering of the sequences using Paracel transcript assembler yielded 7738 unique sequences (redundancy factor of 1.6), demonstrating the excellent normalization achieved by this approach. Twenty-eight ESTs or EST clusters, mostly from later larval stages, had matches to food organisms such as Artemia and were removed from the database. The 7710 remaining unique ESTs consisted of 2556 unique clusters and 5154 singletons. The normalization of the libraries precludes an accurate analysis of transcript abundance, although sequences present multiple times in the normalized libraries presumably correspond to high abundance transcripts. Of the clones that were represented 5 times or more in a given library, the most common were a lectin-like protein, a 14 Kda apolipoprotein, tropomyosin, parvalbumin, and apolipoprotein A1 (Table 2). Parvalbumin, lectin-like protein and the 14 Kda apolipoprotein were each over-represented in two libraries.

**Table 1:** Characteristics of Atlantic halibut normalised cDNA libraries

					Redundancy			
					
	Library	Recom. Eff.	# Clones	Avg. insert size (kb)	2	3	4	5 to 15
Tissue	Gill	95	933	1.4	42	5	0	1
	Head Kidney	94	932	1.4	23	2	0	0
	Intestine	91	910	1.3	45	9	0	0
	Liver	95	906	1.3	69	13	4	3
	Ovary	98	1609	1.5	154	27	6	4
	Skin	97	945	1.4	32	6	1	1
	Spleen	91	898	1.4	29	4	0	0
	Testis	95	1532	1.5	84	11	1	0

Larval	Hatching	96	953	1.6	67	9	1	3
	Mouth-opening	97	950	1.4	39	5	3	5
	Midway to Metamorphosis	97	915	1.6	41	0	1	0
	Premetamorphosis	94	929	1.4	47	3	0	0
	Postmetamorphosis	96	915	1.5	30	5	1	3

The recombination efficiency represents the number of clones containing inserts. The number of clones sequenced, the average insert size in kb (based on analysis of 96 clones), and the number of clones represented more than once (redundancy) for each library are presented.

**Table 2:** Most highly represented clones found in each cDNA library

Library	Gene	Accession #	# hits
Gill	lectin-like protein	AAI15054.1	7
Liver	epoxide hydrolase	NP_957362.1	5
	14 kDa apolipoprotein	CAH57705.1	16
Ovary	ribosomal protein L3	CAF95545.1	5
	transaldolase 1	AAH68191.1	5
	fish eggshell protein	AAS55644.1	5
	unknown EST	AW012936.1	6
Skin	lectin-like protein;	AAI15054.1	7
Hatch	NAD(P)H dehydrogenase quinone 1	ABD43173.1	5
	lipocalin family	CAF97961.1	6
Mouth-opening	parvalbumin beta	AAK63087.1	5
	myosin heavy chain	BAB12571.1	5
	14 kDa apolipoprotein	CAH57705.1	6
	apolipoprotein AI precursor	CAH59609.1	8
	tropomyosin	BAB20881.1	13
Post-metamorphosis	alpha actin 1	AAH45406.1	5
	myosin light chain 2	CAD32552.1	6
	parvalbumin beta	AAK63087.1	10

Genes that were represented more than five times in a given cDNA library are listed.

Screening of EST sequences for short tandem repeats (2 – 5 bp) identified 129 that contained microsatellite sequences. Of these, 60 had 2 bp repeats (mostly GT or GA), 58 had 3 bp repeats, 7 had 4 bp repeats and 4 had 5 bp repeats. Sixty of these loci were polymorphic and were incorporated into our halibut linkage map (D. Reid, C. Smith, D. Martin-Robichaud, M. Reith, unpublished). All EST data have been deposited in GenBank (accession numbers EB029285–EB041700 &EB080851–EB080975), and preliminary annotations are available on the Pleurogene website [35].

### Annotation

All ESTs and clusters were initially annotated with AutoFACT, which carries out a series of Blast analyses to identify the EST and attempts to assign function and/or pathway information. AutoFACT provided product or gene names for 4786 sequences (62%) and 254 additional sequences had domain names associated with them (Table 3). Of the remaining sequences, 28 matched ribosomal RNAs, 824 matched unassigned proteins, 802 matched unknown ESTs, and 1016 had no database hits ("unclassified").

**Table 3:** Classification of Atlantic halibut unique sequences

Classification	Classification Method	GO source	Number of Sequences			
Unclassified	No BLAST hit		1016			
Ribosomal RNA	rRNA hit		28			
Unassigned protein	BLAST hit >e-10 to unknown protein		824			
Unknown EST	BLAST hit >e-10 to unknown EST		802			
Functionally annotated protein	BLAST hit >e-10 to known protein		5040			
	Informative terms			4786		
	Domain name-containing protein			254		
	Gene Ontology (107)				3878	
		AutoFACT				1640
		Goblet				1736
		InterPro				605
	KEGG (185)				578	
	COG (22)				1191	

Total			7710	5040		

Numbers of sequences associated with AutoFact classification. The method of classifying functionally annotated proteins is indicated and the numbers of categories identified by GO, KEGG and COG are given in brackets. GO classifications were obtained using Goblet and Interpro information as well as AutoFACT.

**Figure 2:**
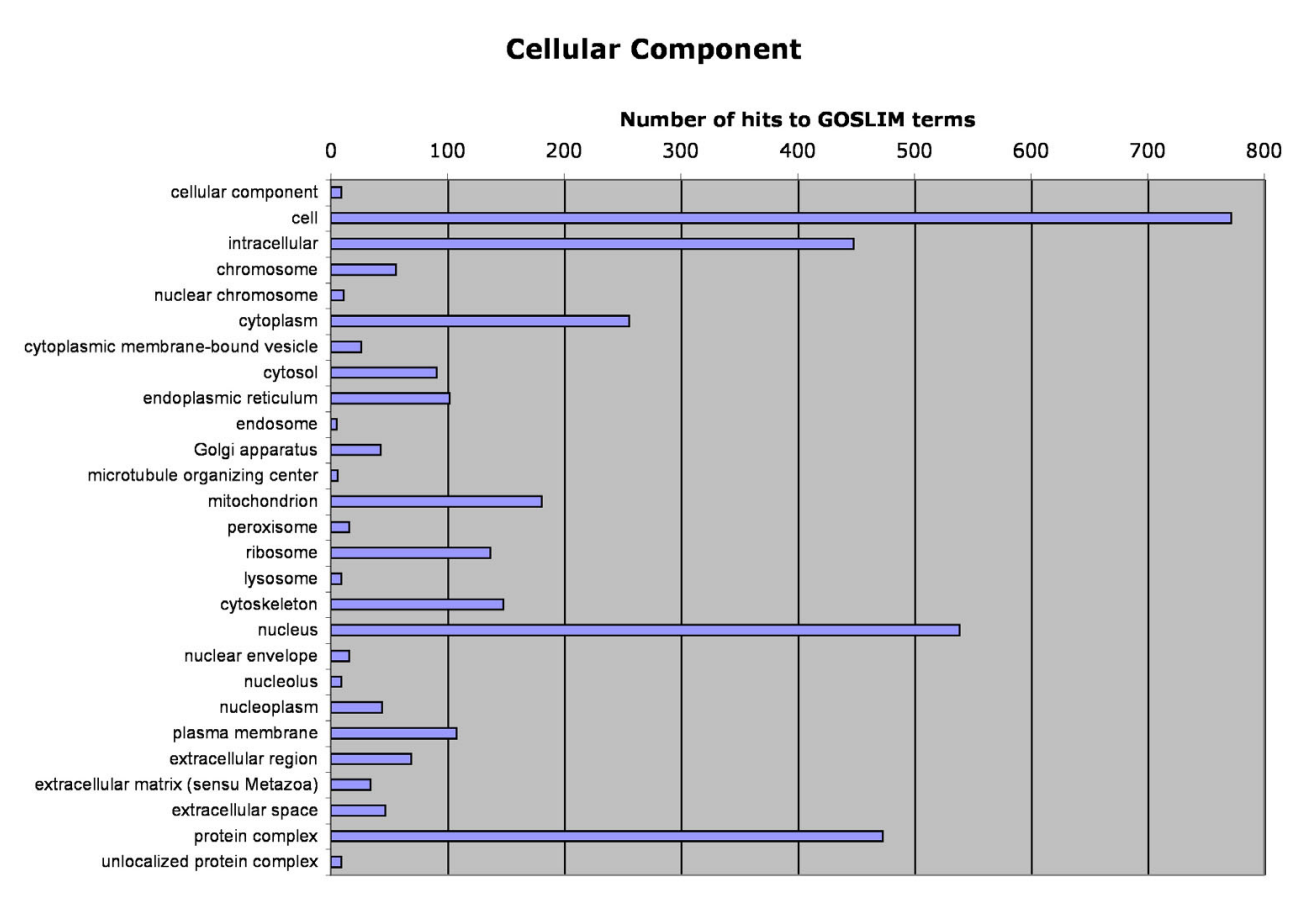
Classification of Atlantic halibut unique sequences according to Gene Ontology (GO) category: cellular component.

**Figure 3:**
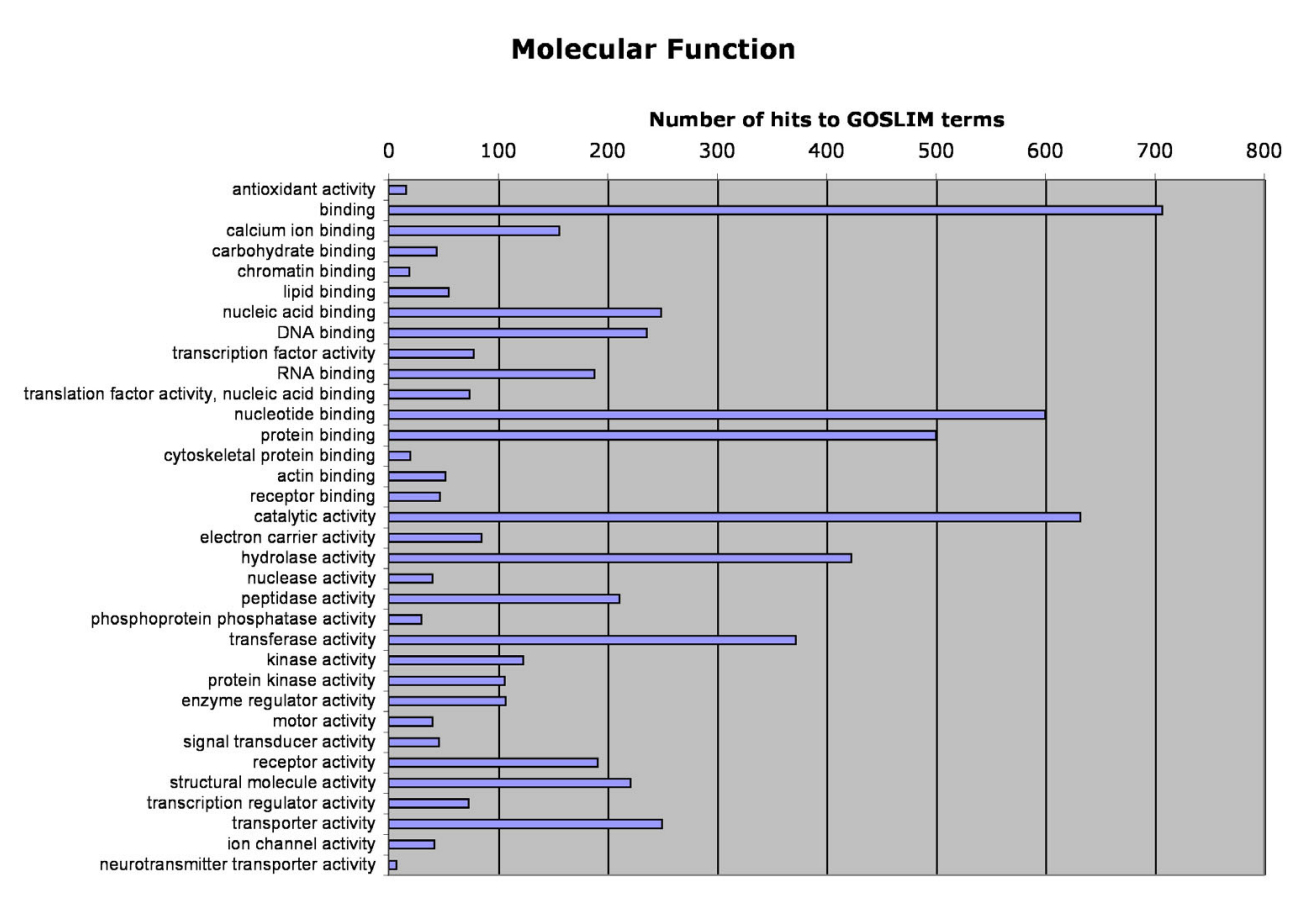
Classification of Atlantic halibut unique sequences according to Gene Ontology (GO) category: molecular function.

**Figure 4:**
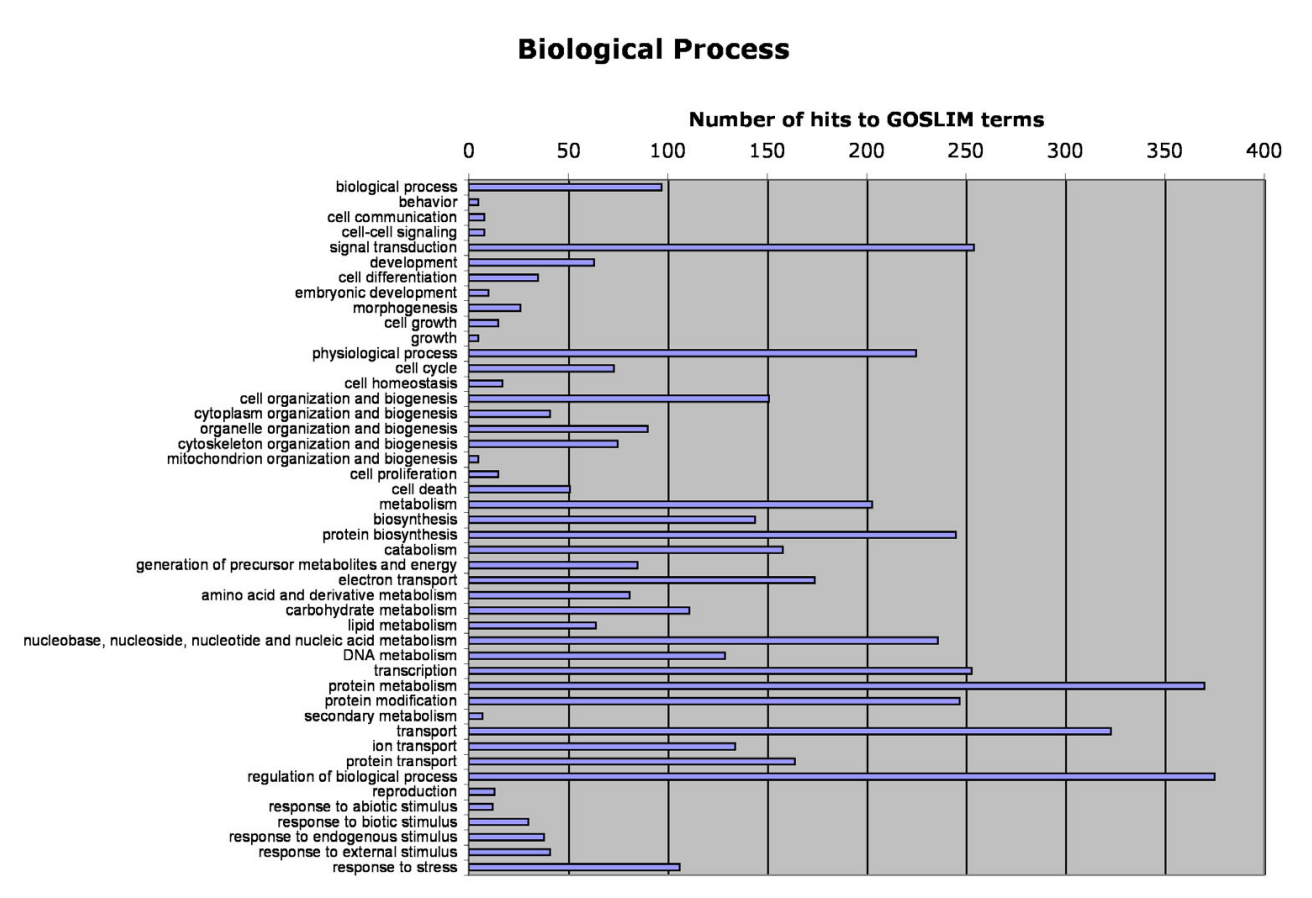
Classification of Atlantic halibut unique sequences according to Gene Ontology (GO) category: biological process.

Of the 5040 functionally annotated and domain name-containing sequences, Gene Ontology (GO) classifications were found for 3878 using AutoFACT, Goblet and InterPro. The distribution of sequences among GO classifications are represented in Figures 2, 3, 4. Sequences with GO terms corresponding to cellular component fell into 27 categories (Figure 2), molecular function into 34 categories (Figure 3) and biological process into 46 categories (Figure 4). Clusters of Orthologous Groups (COG; [36]) categories were determined for 1191 sequences, and Kyoto Encyclopedia of Genes and Genomes (KEGG; [37]) categories were found for 578 sequences. Sequences assigned to COG fell into 22 categories and those assigned to KEGG fell into 185 pathways (Table 3).

We also performed a similar analysis on the 4110 partially annotated Atlantic halibut sequences deposited in GenBank by other groups. After assembly, a unigene set of 2337 sequences was obtained of which 40 were rRNA, 781 were unclassified, 80 matched unassigned proteins and 1436 were informative annotations. However, of the informative annotations, 531 fell into five clusters: nuclease diphosphate kinase B (73), cytochrome c oxidase subunit III (109), cytochrome c oxidase subunit II (182), cytochrome c oxidase subunit I (62) and cytochrome b (105). Similarly, of the 788 sequences that received COG annotations, over half (487) were associated with energy production and conversion, and were predominantly of mitochondrial origin.

When the complete EST set of 12675 sequences was searched for GO terms using the GOSLIM classification, two libraries were substantially more enriched in GOSLIM terms than others: the ovary library had 4496, and the testis library had 3371 hits to GOSLIM terms, respectively. Some of this enrichment can be explained by the increased number of ESTs sequenced from these libraries (50%), but even taking this into account, there are still many more GOSLIM terms in each of these libraries than the other tissue-specific libraries, which have between 1017 (gill) and 1871 (liver) terms. Of the larval libraries, that from the mouth-opening stage had 2415 hits to GOSLIM terms. Interestingly, the library constructed from larvae midway to metamorphosis had only 443 hits to GOSLIM terms. The remainder of the libraries had between 1444 and 1788 GOSLIM terms.

### Functional categories of cDNAs

The functional categories included in the COG and GO classification schemes are not entirely congruent and thus not directly comparable. Broadly speaking, the COG classification gave a good overview of the cellular processes represented in the EST library. As can be seen from Table 4, approximately one third of the ESTs are associated with a COG category related to transport, metabolism and energy, 44% of the ESTs are associated with nucleic acid processes, and 4% of the ESTs are associated with cell structure. The remainder of the classified ESTs represent genes involved in signal transduction, defense and intracellular trafficking. KEGG annotations were found for 578 different sequences, the most common pathways represented are shown in Table 5.

**Table 4:** Classification of Atlantic halibut unique sequences according to COG

Category	#	%
Amino acid transport and metabolism	72	6.05
Carbohydrate transport and metabolism	61	5.12
Coenzyme transport and metabolism	22	1.85
Inorganic ion transport and metabolism	27	2.27
Lipid transport and metabolism	42	3.53
Nucleotide transport and metabolism	43	3.61
Secondary metabolites biosynthesis, transport and catabolism	13	1.09
Energy production and conversion	128	10.75
Total metabolism and energy	408	34.26
Cell cycle control, cell division, chromosome partitioning	10	0.84
Chromatin structure and dynamics	14	1.18
Replication, recombination and repair	41	3.44
RNA processing and modification	6	0.50
Transcription	51	4.28
Translation, ribosomal structure and biogenesis	188	15.79
Posttranslational modification, protein turnover, chaperones	211	17.72
Total nucleic acid processes	521	43.74
Cell wall/membrane/envelope biogenesis	6	2.14
Cytoskeleton	40	3.36
Total cell structure	46	3.86
Intracellular trafficking, secretion, and vesicular transport	37	3.11
		
Signal transduction mechanisms	33	2.77
		
Defense mechanisms	4	0.34
		
General function prediction only	132	11.08
Function unknown	10	0.84
Total	1191	100.00

Categories were broadly grouped into metabolism and energy, nucleic acid processes and cell structure. The number (#) and percent (%) of ESTs in each category is shown.

**Table 5:** Most commonly represented KEGG classifications of Atlantic halibut unique sequences

Category	#	%
Oxidative phosphorylation	101	17.5
Ribosome	58	10.0
Purine & pyrimidine metabolism	41	7.1
Proteasome	27	4.6
Cell communication	21	3.6
SNARE interactions and vesicular transport	17	2.9
Arginine and proline metabolism	16	2.8
Transcription factors	16	2.8
Complement and coagulation cascades	13	2.2
Glycan structures	12	2.1
Arachidonic acid metabolism	11	1.9

Categories with more than ten sequences out of a total of 578 are listed. The number (#) and percent (%) of ESTs in each category is shown.

## Discussion

To improve our understanding of flatfish biology and the problems associated with their development and rearing, a comparative genomics program focusing on Atlantic halibut and Senegal sole (*Solea senegalensis*) has been initiated (see [35]). As a prelude to construction of a DNA microarray, the EST survey reported here has been carried out.

Two previous EST surveys have been conducted in Atlantic halibut: one from a study of the effect of vaccination [28], resulting in approximately 1000 sequences, and a second from an investigation of muscle somite formation [38], resulting in approximately 4250 sequences. The study reported here greatly enriches the genomic resources for this commercially important flatfish by adding more than 12,000 ESTs to the partially annotated sequences that had already been deposited.

Over 5000 of the 7710 unique transcripts represented by the ESTs have been functionally annotated. These annotations and the development of a searchable database containing all of the information associated with each EST add enormous value to such a study. The main categories of genes represented in the ESTs are involved in binding, catalytic activity, transport, metabolism, response to stimuli, signal transduction, nucleic acid processes, and cellular biogenesis. Again, this adds substantially to the Atlantic halibut sequences from other research groups, many of which were of mitochondrial origin, and which contained slightly over 900 informative AutoFACT annotations.

With only modest resources for EST sequencing available, we choose to normalize our cDNA libraries so as to maximize the number of different ESTs sequenced. The normalization method used (Evrogen Trimmer kit) was very effective at reducing the number of highly expressed cDNAs. Since the libraries are well-normalised (redundancy factor of only 1.5), it is not possible to gain insights into the actual abundance of different types of transcripts; however, the enrichment in GO terms in the reproductive tissues and one of the larval libraries indicates that these libraries represent a broad diversity of transcripts, indicative of the high metabolic and proliferative characteristics of ovary and testis tissues. Larvae at the early mouth-opening stage of development are also undergoing tremendous metabolic changes as they transition from the yolk-sac to first-feeding stages. On the other hand, the library made from larvae midway to metamorphosis has very few GO terms associated with it, possibly because a large number of genes associated with the unusual metamorphic process in flatfish have not yet been described.

A number of ESTs were restricted to only a single tissue library and as such, may be good tissue-specific markers for *in situ* hybridisation and aid in tracking the appearance of different tissues during development [39,40]. For example, ESTs for a renal organic ion transporter and nephrosin are only found in the head kidney library. Several transporters (for amino acids and various solutes), binding proteins (for lipid, sterol and lipoproteins) as well as digestive enzymes (elastases, peptidase, aminopeptidase N, carboxypeptidase B, triglyceride lipase) are only found in the intestine library. Various complement components, apolipoproteins, fatty acid binding protein, alpha-2-macroglobulin and biliverdin reductase A are only present in the liver library. A single EST unique to spleen was identified – metaxin 2, similar to the von Willebrand clotting factor. Unique to the ovary library were a number of ESTs specific to reproduction – zona pellucida protein, vitelline envelope protein, chorion protein, choriolytic enzyme, aquaporin, alveolin, estrogen receptor binding protein and luteinizing hormone beta. Similarly, the testis library uniquely contained ESTs specific to reproduction – spindlin protein C, testis intracellular mediator protein, a cysteine and glycine-rich protein, and periostin. The gill library uniquely contained two ESTs involved in chloride transport and the skin uniquely contained keratin, epithelial membrane protein-3, epiplakin and dermatopontin.

The liver, head kidney and spleen libraries proved to be an excellent source of genetic information concerning hematopoiesis and immune function in this fish. The head kidney is the major site of hematopoiesis in fish and an EST survey of zebrafish kidney revealed many insights into this process in fish [41]. From our Atlantic halibut EST survey, many complement components, immune type receptors, lectins, defense proteins, MHC I and II components, cytokines, chemokines as well as signal transduction molecules and transcription factors involved in expression of immune genes were identified. It should be noted that ESTs for components of the immune system were also found in other tissue libraries, particularly those exposed to the environment such as skin and gill; these arose from circulating or resident immune cells in these tissues. This new sequence information will greatly enhance our understanding of the immune system of flatfish and provide molecular tools for further studying disease resistance.

The identification of microsatellite sequences in the Atlantic halibut ESTs will aid in the completion of a genetic linkage map of Atlantic halibut that is currently being constructed. Since these microsatellites are linked to genes, they are useful as Type I markers.

## Conclusion

The addition of over 7700 ESTs, of which 5040 are functionally annotated, significantly enhances the genomic tools available for non-model fish species. Given the high degree of sequence similarity between flatfish species, the Atlantic halibut ESTs will be of great interest to the flatfish researchers in general, as well as the halibut aquaculture research community. The publicly accessible, searchable database also adds substantial value to the genomic data generated in this study. This EST survey has provided a number of microsatellite markers that have been placed on the Atlantic halibut genetic linkage map (Reith, pers. comm.) as well as probes for cellular localisation studies by *in situ* hybridisation. It has also laid the groundwork for the design and construction of a microarray for studying gene expression under different environmental conditions to better understand the impact of nutrition, stress, and environmental conditions on aquaculture production.

## Methods

### Fish rearing and sampling

Larvae were reared at Scotian Halibut Limited (Clarks Harbour, NS, Canada) in constant light (approximately 1000 lux at the surface) in 7 m^3 ^tanks with flow-through salt water (32 ppt) maintained at 11 ± 0.2°C using a heat exchanger. Larvae were fed Artemia until weaning onto artificial feed at 65 DPH. The ages and sizes of the larvae at the different stages were as follows: hatching (1 dph; 10 mm), mouth-opening (21 dph;15 mm), midway to metamorphosis (64 dph; 20 mm), premetamorphosis (91 dph; 25 mm), and post-metamorphosis (104 dph; 30–35 mm).

Livers were sampled from one male (104 cm; 18 kg) and one female (130 cm; 31 kg) adult broodstock maintained at St. Andrews Biological Station, St. Andrews, NB, Canada. All other tissues were sampled from 3 male and 3 female immature fish (672–945 g) reared at the Institute for Marine Biosciences Marine Research Station (Sambro, NS, Canada). Immediately before sampling, fish were transferred to a bucket containing an overdose of TMS-Aqua MS-222 (Syndel, Vancouver, BC, Canada). Ovaries from 3 females were pooled, as were testes from 3 males. Gill, head kidney, intestine, skin, and spleen samples from each group of 6 fish were pooled, and all tissue samples preserved in RNALater (Ambion, Austin, TX, USA), and stored at -80°C until use. Larvae were pooled and preserved in RNALater, and stored at -80°C until use. All animal procedures were approved by the NRC Institute for Marine Biosciences Animal Care Committee.

### cDNA library construction

For liver, mRNA was extracted from one female and one male halibut using the FastTrack kit (Invitrogen, Burlington, ON, Canada) and equal amounts of mRNA were combined. For spleen, total RNA was extracted from pooled tissues using Trizol Reagent (Invitrogen). For other tissues, mRNA was extracted from pooled tissues using the Micro-FastTrack kit (Invitrogen). For larval libraries, RNA was extracted from pooled samples (~20 larvae each for post-hatch and mouth-opening stages, 15 larvae for midway to metamorphosis, and 5 larvae each for pre- and post-metamorphosis) using Trizol Reagent. All RNA isolation kits were used according to the manufacturer's protocols.

First strand cDNA was prepared from 0.25–0.4 μg mRNA or 2 μg total RNA using the Creator SMART cDNA method (Clontech, Palo Alto, CA, USA) and PowerScript reverse transcriptase (Clontech). The CDS-3M adaptor, included in the TRIMMER-DIRECT kit (Evrogen, Moscow, Russia), was used instead of the SMART CDSIII primer. cDNA was amplified by LD-PCR according to the Creator SMART cDNA method (Clontech) using the 5' PCR primer as the forward and reverse primer. The optimal number of cycles to yield sufficient cDNA for normalisation, but remain within the exponential phase of amplification, was determined by analysing aliquots of the PCR reaction after every second cycle on agarose gels. In all cases, sufficient cDNA was obtained in 18 cycles or less, ensuring even the rarest messages were represented. Amplified cDNA was purified using the QIAquick PCR purification kit (Qiagen, Valencia, CA, USA), quantitated using a NanoDrop^® ^ND-1000 spectrophotometer (NanoDrop^® ^Technologies Inc, Wilmington, DE, USA) and normalized using the TRIMMER-DIRECT protocol (Evrogen). After digestion with SfiI, products smaller than 500 bp were removed using the Chroma Spin-400 column column as described in the Creator SMART protocol.

The resulting cDNAs were directionally cloned into the SfiI sites of pDNR-LIB (Clontech) and transformed into ElectroMAX DH10B T1 phage-resistant cells (Invitrogen) by electroporation using the Cell Porator and Voltage Booster system (Gibco BRL). The Cell Porator settings were 400 V, 330 μF capacitance, low Ω impedance and fast charge rate, and the Voltage Booster was set at 4 kΩ. For each library, 10^6 ^primary transformants were amplified by the semi-solid amplification method described in Stratagene's pBluescript XR cDNA library construction kit manual. Randomly picked clones (96 from each library) were screened for insert size by protoplasting [42] or by PCR using the M13 forward and reverse primers flanking the multiple cloning site of the vector.

### Sequencing

Individual bacterial colonies were picked into 96- or 384-well plates containing LB/glycerol using the QPix colony picker (Genetix Ltd., New Milton, Hampshire, UK). A 96-well test plate was prepared from each library for sequencing and if the quality of the library was good, clones were sequenced from two additional 96-well and two 384-well plates, giving a total of 1056 reads. Two additional 384-well plates were sequenced from each of the ovary and testis libraries. Plates were incubated overnight at 37°C. The resulting bacterial suspensions were inoculated into lysis buffer and denatured at 95°C for 5 minutes. DNA from each clone was amplified using TempliPhi™ DNA polymerase (GE Healthcare, Baie d'Urfe, QC, Canada) according to manufacturer's instructions. DNA sequencing was performed using ET terminator chemistry (GE Healthcare) in the 5' direction (primer sequence GGCCGCATAACTTCGTATAGC). Reactions were processed using Sera-Mag™ magnetic carboxylate-modified microparticles (Seradyn™, Indianapolis, IN, USA) to remove excess fluorescent terminators before loading onto GE Healthcare MegaBACE 4000 capillary DNA sequencers. Clones from each library were replicated into glycerol stocks and stored at -80°C.

### ESTs and annotation

ESTs were clustered using Paracel Transcript Assembler 3.0 (Paracel Inc., Pasadena, CA), which is based on the CAP4 clustering algorithm [43]. Annotation was performed using AutoFACT [44] and the default parameters with UniProt's UniRef90, NCBI's nr, KEGG, COG, PFAM, LSU, SSU and, for contigs, est_others. AutoFACT summary results are stored in our database [35]. Each sequence has one or more AutoFACT results associated with it. Each AutoFACT result is related to the most informative BLAST hit from each of the queried set of databases. Those hits are also stored in the Pleurogene database. GO annotations associated with either the contributing hits or the AutoFACT results are stored in a separate table.

Due to the low level of GO annotation obtained with AutoFACT (1640 sequences out of 7710), we chose to run the unannotated sequences through Goblet [45] using the vertebrate database and a BLAST cutoff of e-10. This increased the annotation level by 1736 sequences. Because the default criteria for a match in Goblet is lower than in AutoFACT and these sequences had already passed through AutoFACT, these GO annotations should be regarded as less reliable. GO terms were also identified for an additional 502 sequences by searching InterPro. Functionally annotated ESTs with GO annotations were classified using GOSlim and each category with more than 5 hits was plotted. Individual ESTs in each library (13,000 total) were also searched for GOSLIM terms and compiled by library.

### Identification of microsatellite sequences

Short tandem repeats (2 – 5 bp) were detected using Tandem Repeats Finder [46].

## Authors' contributions

SED and MER conceived of and designed the project. LCK constructed cDNA libraries. JK and MER carried out bioinformatic analyses. SED wrote the manuscript. MER and SED edited the manuscript. All authors read and approved the manuscript.
